# Systemic Dietary Hesperidin Modulation of Osteoclastogenesis, Bone Homeostasis and Periodontal Disease in Mice

**DOI:** 10.3390/ijms23137100

**Published:** 2022-06-26

**Authors:** Vinícius de Paiva Gonçalves, Marta Liliana Musskopf, Angeliz Rivera-Concepcion, Christina Yu, Sing Wai Wong, Stephen A. Tuin, Yizu Jiao, Cristiano Susin, Luís Carlos Spolidorio, Patricia Almeida Miguez

**Affiliations:** 1 Division of Comprehensive Oral Health, School of Dentistry, Adams School of Dentistry, University of North Carolina at Chapel Hill, Chapel Hill, NC 27599-7455, USA; viniciusodonto2007@hotmail.com (V.d.P.G.); singwaiw@usc.edu (S.W.W.); csusin@email.unc.edu (C.S.); 2 Adams School of Dentistry, University of North Carolina at Chapel Hill, Chapel Hill, NC 27599-7455, USA; mlmusskopf@unc.edu (M.L.M.); arivcon@email.unc.edu (A.R.-C.); christinayuu36@gmail.com (C.Y.); satuin@gmail.com (S.A.T.); yizujiao@live.unc.edu (Y.J.); 3 Department of Physiology and Pathology, Araraquara School of Dentistry, Araraquara 14801-903, SP, Brazil; luis-carlos.spolidorio@unesp.br

**Keywords:** flavonoids, hesperidin, osteoclasts, bone resorption, periodontal disease, bone homeostasis

## Abstract

This study aimed to evaluate the effects of hesperidin (HE) on in vitro osteoclastogenesis and dietary supplementation on mouse periodontal disease and femoral bone phenotype. RAW 264.7 cells were stimulated with RANKL in the presence or absence of HE (1, 100 or 500 µM) for 5 days, and evaluated by TRAP, TUNEL and Western Blot (WB) analyses. In vivo, C57BL/6 mice were given HE via oral gavage (125, 250 and 500 mg/kg) for 4 weeks. A sterile silk ligature was placed between the first and second right maxillary molars for 10 days and microcomputed tomography (μCT), histopathological and immunohistochemical evaluation were performed. Femoral bones subjected or not to dietary HE (500 mg/kg) for 6 and 12 weeks were evaluated using μCT. In vitro, HE 500 µM reduced formation of RANKL-stimulated TRAP-positive(+) multinucleated cells (500 µM) as well as c-Fos and NFATc1 protein expression (*p* < 0.05), markers of osteoclasts. In vivo, dietary HE 500 mg/kg increased the alveolar bone resorption in ligated teeth (*p* < 0.05) and resulted in a significant increase in TRAP+ cells (*p* < 0.05). Gingival inflammatory infiltrate was greater in the HE 500 mg/kg group even in the absence of ligature. In femurs, HE 500 mg/kg protected trabecular and cortical bone mass at 6 weeks of treatment. In conclusion, HE impaired in vitro osteoclastogenesis, but on the contrary, oral administration of a high concentration of dietary HE increased osteoclast numbers and promoted inflammation-induced alveolar bone loss. However, HE at 500 mg/kg can promote a bone-sparing effect on skeletal bone under physiological conditions.

## 1. Introduction

Bone is a remarkably dynamic and active tissue, undergoing constant renewal in response to mechanical, nutritional, and hormonal influences [1]. Under physiologic conditions, the bone remodeling is supported by a balance between the coupled processes of bone resorption and bone formation, which is also influenced by local factors (including cell–cell interactions and extracellular matrix molecules) and orchestrated by osteocytes and bone lining cells [1,2]. The regulated coupling of resorption by osteoclasts to new bone formation by osteoblasts is required for proper growth, remodeling, and skeletal maintenance [1,3,4,5].

Osteoclasts are the major cells responsible for bone resorption, and activated osteoclasts release proteolytic enzymes which destroy connective tissues in bones, and also secrete acids that dissolve the mineral portion of bones [6]. Bone resorption is a basic physiologic process that is central to the understanding of bone homeostasis and many pathologies [1], and excessive resorption found in pathological conditions such as periodontitis is the most common oral manifestation of alveolar bone destruction [1,7,8].

Periodontitis is defined as a multifactorial inflammatory disease that affects the supporting structures of the teeth [9], due to the immune response of susceptible hosts to the action of microorganisms [10]. Alveolar bone loss is a hallmark of periodontitis progression and bone destruction is mediated by the host immune and inflammatory response to the microbial challenge [1]. Osteoclasts differ from monocyte/macrophage precursors under the regulation of cytokines, macrophage colony-stimulating factor, receptor activator of nuclear factor kappa B (RANK) ligand (RANKL), and osteoprotegerin (OPG). The pathogenic processes of destructive inflammatory periodontal diseases are instigated by subgingival plaque microflora and factors such as lipopolysaccharides (LPS) derived from specific pathogens [1]. The innate immune response primarily started by neutrophils [1] will lead to the activation of T and B cells which initiate the adaptive immune response via regulation of the Th1–Th2–Th17 regulatory axis. In summary, Th1-type T lymphocytes, B cell macrophages, and neutrophils promote bone loss through upregulation of proinflammatory mediators and activation of the RANKL expression pathways [1].

Strategies to prevent periodontal tissue breakdown or serve as coadjuvants to mechanical treatment of periodontitis intended to prevent or halt the progression of periodontal bone resorption are still needed due to variability in outcomes of periodontal treatments (mechanical debridement with and without antibiotics) [11]. In this context, adjuvant therapies that promote improvement of the results of conventional periodontal treatment have been proposed, such as the use of herbal medicines [12,13,14,15,16]. Natural compounds derived from the group of flavonoids such as hesperidin (HE) have been associated with the reduction of inflammatory cytokines in vitro and inflammatory responses in vivo in animals challenged with bacteria and bacterial products such as LPS [17,18]. Further, flavonoids promote osteoblast differentiation [19], increase the expression of OPG and reduce the production of RANKL [20], suggesting potential for periodontitis modulation. HE represents, within the class of flavonoids, the most researched compound on skeletal bone health and metabolism, as described by Sacco et al. (2013) [21]. The beneficial effects of HE on bone tissue appear to be reduction of bone resorption [21,22] and promotion of bone formation [23,24]. It has also been suggested that HE has an additional protective effect on bone by modulating the production of inflammatory mediators [22]. Recently, our research evaluating the direct effect of HE on osteogenesis showed that HE has a positive role in mineralized tissue formation via not only promotion of osteoblast cell differentiation, but also improvement of matrix organization and matrix-to-mineral ratio, providing a basis to the idea that it could be a potential adjunct in regenerative and maintenance-driven bone therapies [25].

The HE effects associated with an experimental periodontal disease model were first published in 2019 [26], and the authors reported an ameliorative and dose-dependent effect of HE (at 75 and 150 mg/kg concentrations) on ligature-induced periodontitis in rats, and showed that HE prevented the progression of ligature-induced bone loss and effectively inhibited the expression of gingival pro-inflammatory cytokines [26]. Compiling evidence about HE’s effects on bone supports the hypothesis that the compound can be a potential regulator of bone metabolism in pathological inflammatory and/or resorptive conditions, making it a promising adjuvant in periodontitis management.

We studied osteoclast differentiation, periodontal disease establishment and bone homeostasis in the presence of various doses of HE in an effort to further understand the effect of HE in inflammatory vs. non-inflammatory bone homeostasis.

## 2. Results

### 2.1. Adverse Events

**Figure 1:**
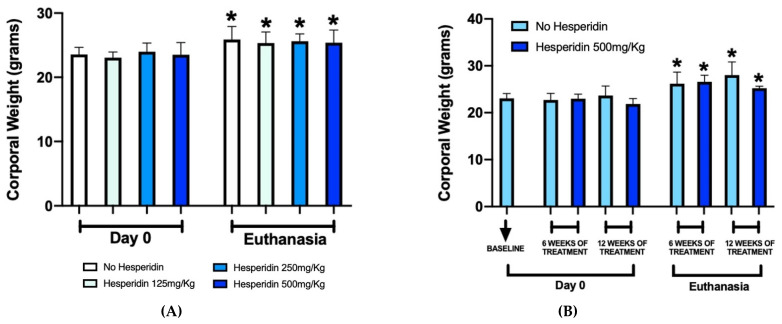
Corporal weight of mice before experiments (Day 0) and at euthanasia. The graphs show a uniform increase in corporal weight in both periodontal disease (**A**) and femur (**B**) mouse models at euthanasia compared to Day 0. * *p* < 0.05 compared to the respective group at Day 0.

Ligature placement or systemic HE administration did not promote any behavioral change, physical issues (e.g., skin lesions, hair loss), detrimental weight loss or feeding impairment (Figure 1).

### 2.2. HE Treatment Did Not Promote RAW 264.7 Cell Apoptosis

**Figure 2:**
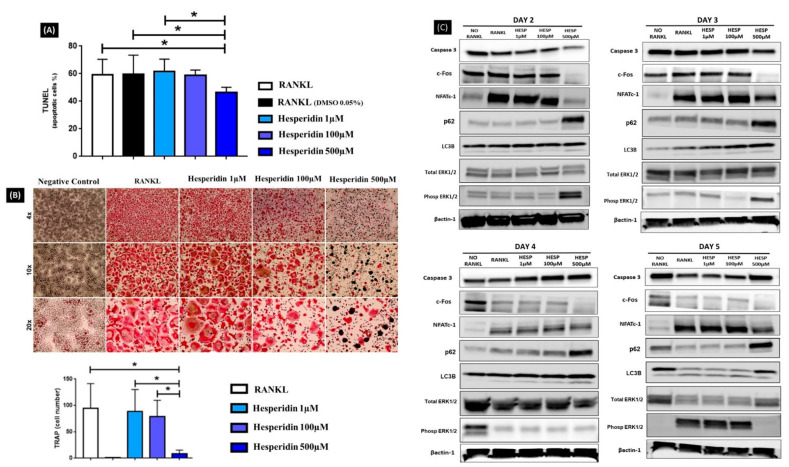
Overview of in vitro RANKL-induced osteoclastogenesis. RAW 264.7 cells were stimulated by RANKL with or without hesperidin treatment for 5 days. There were no differences in the percentage of apoptotic cells after 5 days of in vitro differentiation compared to RANKL control groups as measured by the TUNEL assay, except for the 500 µM HE group (**A**). (**B**) Representative images demonstrate cells differentiated in osteoclasts stained in pink by TRAP assay, 5 days post RANKL stimulation (4×, 10× and 20× magnification). Lower graph represents the average number of osteoclasts (TRAP+) quantified per group including no RANKL addition (no bar/no cells observed). Cells with 3 or more nuclei are considered osteoclasts. * *p* < 0.05 compared to the 500 µM hesperidin group. Hesperidin significantly affected differentiation of osteoclasts in a dose-dependent manner. (**C**) Protein expression for Caspase 3, c-Fos, NFATc1, p62, LC3, Total ERK ½, Phosphorylated ERK ½ and Beta-Actin1 was assessed by Western blot (WB) on days 2, 3, 4 and 5 post-RANKL treatment from the sample protein lysates. Results showed influence of hesperidin on autophagy markers LC3 to LC3-IIB (upper and lower bands in LC3B row, respectively), p62 and ERK ½ with day 5 showing most clear autophagy inhibition effect via accumulation of LC3, marked increase in p62 and absence of ERK ½ phosphorylation (WBs were done in triplicates and representative images displayed).

RANKL-stimulated cells showed a survival percentage of approximately 60% after treatment with DMSO and no difference was found with HE concentrations at 1 and 100 µM. Treatment with the highest concentration of HE (500 µM) significantly reduced the overall percentage of apoptotic cells (*p* < 0.05), although when normalized to total number of cells, there was no difference (Figure 2A).

### 2.3. Osteoclast Differentiation Was Compromised with Treatment of HE

RANKL stimulation promoted expressive RAW 264.7 cells differentiation into osteoclasts, as observed by positive TRAP staining. The number of differentiated cells under treatment with HE at 1 and 100 µM was maintained when compared to the control group only stimulated with RANKL. Significantly reduced formation of RANKL-stimulated TRAP-positive(+) multinucleated cells was observed after treatment with the highest HE concentration (500 µM) (*p* < 0.05). The negative control group (no RANKL) did not present osteoclast differentiation (Figure 2B).

### 2.4. HE Treatment Downregulated c-Fos and NFATc1 Expression during In Vitro Osteoclastogenesis and Was Associated with Changes in Autophagy Markers

On the WB (Figure 2C) of RANKL-stimulated differentiation, the c-Fos protein expression was most noticeably downregulated under HE treatment at the highest concentration (500 µM). The expression of NFATc1, a master regulator of osteoclastogenesis, was observed at every timepoint but decreased with addition of 500 µM of HE to media. Conversely, HE at 500 µM was associated with a high p62 expression at every timepoint as well as increased LC3 expression at days 4 and 5, although LC3-IIB conversion (lower band below LC3, a marker of autophagy) was not particularly increased. ERK ½ phosphorylation was markedly reduced at day 5 for the higher concentration of HE (*p* < 0.05, *n* = 3).

Caspase 3, a marker of apoptosis, remained the same in the negative control, RANKL alone and HE-treated groups throughout in vitro osteoclastogenesis, except for the higher dose of HE which appeared to increase Caspase 3 at day 5.

### 2.5. Treatment with HE at 500 mg/kg Was Associated with Increased Ligature-Induced Bone Resorption

**Figure 3:**
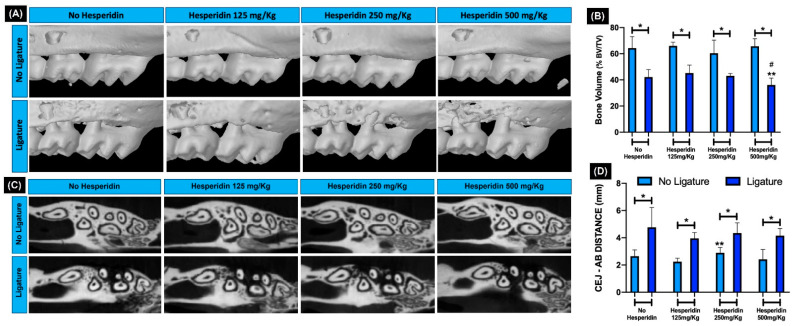
Two and three-dimensional analysis of alveolar bone. (**A**) 3D and (**C**) 2D rendering of maxillae showing bone levels. (**B**) Graph expresses the average percentage of bone volume (BV) to total volume (TV) in each experimental group analyzed using µCT, showing significant bone loss promoted by ligature in all groups. Treatment with hesperidin at 500 mg/kg significantly decreased bone volume compared to the lowest concentrations of hesperidin. * *p* < 0.05 comparing group with and without ligature. ** *p* < 0.05 compared to the group ** 125 mg/kg + ligature and # 250 mg/kg + ligature. The 3D (**A**) and 2D (**C**) representative images show alveolar bone integrity verified in all groups of animals that did not receive ligature, while in the other groups subjected to experimental periodontitis, bone resorption can be observed. Correlated to these findings, the linear measurement of cemento-enamel junction (CEJ) distance showed a higher bone loss in the ligature groups (**D**). * *p* < 0.05 comparing group with and without ligature. ** *p* < 0.05 comparing no ligature groups HE 125mg and 250 mg/kg.

Two and three-dimensional evaluation by µCT (Figure 3A–D) showed a significant decrease in alveolar bone volume in all animals with ligature-induced periodontitis in comparison with no-ligature groups (Figure 3B, * *p* < 0.05). However, within HE-treated groups, supplementation at 500 mg/kg significantly decreased bone volume in ligated animals compared to both lower concentrations of HE (Figure 3B, **,# *p* < 0.05). With the linear measurement method of cemento-enamel junction (CEJ)-alveolar bone crest (ABC) distance, there was higher bone loss in the ligature groups compared to no ligature (* *p* < 0.05); however, bone loss was not further increased with any of the HE treatments compared to HE (Figure 3D).

### 2.6. HE Did Not Inhibit Severe Periodontal Tissue Breakdown and the Marked Inflammatory Gingival Infiltrate Induced with Ligature

**Figure 4:**
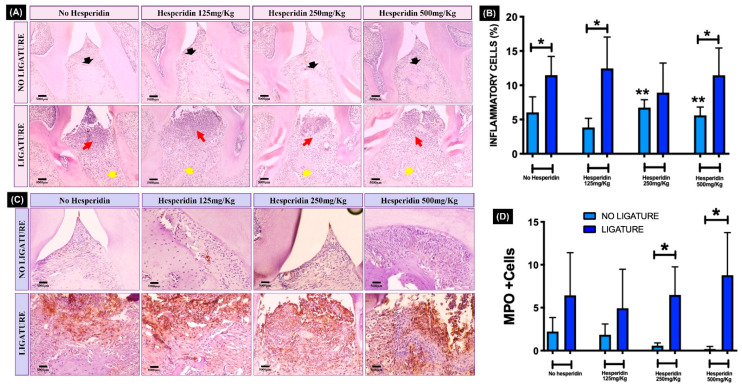
Histopathological and stereometric evaluation of the periodontal tissues (H&E and immunohistochemistry). (**A**) In the representative images from each group, black arrows indicate the integrity of alveolar bone crest in the groups of animals that did not receive ligature, while marked bone resorption (yellow arrows) can be seen in the ligature groups. Red arrows indicate the presence of clear inflammatory gingival infiltrate in all groups with experimental periodontitis confirmed by stereometry (**B**) showing a significant increase in inflammatory cells compared to the no-ligature groups. * *p* < 0.05 comparing ligature and no-ligature groups. ** *p* < 0.05 hesperidin 250 and 500 mg/kg compared to lower dose of hesperidin and no ligature. Complementary to these findings, the myeloperoxidase (MPO)-positive cells, stained in brown via immunohistochemistry (**C**), were increased in all ligature groups and were significantly increased for 250 and 500 mg/kg hesperidin-treated ligated mice compared to their respective no ligature counterpart (* *p* < 0.05) (**D**). Photomicrographs—20× magnification.

Histopathological evaluation of the periodontal structures showed a large and marked tissue breakdown observed in all groups subjected to ligature (Figure 4A). The bone resorption visualized by hematoxylin and eosin (H&E) confirmed the findings observed by µCT analysis.

In the no-ligature groups, with or without HE treatment, the ABC was at typical distance from the CEJ, with normal integrity of the alveolar bone crest and absence of noticeable gingival inflammation. For HE 250 and 500 mg/kg, quantification of inflammatory cells showed statistically significant increase compared to HE 125 mg/kg. As expected, with alveolar bone loss, the presence of a statistically significant amount of inflammatory cell gingival infiltrate was also observed in all ligature groups compared to no ligature (Figure 4B). HE treatment was not able to prevent or reduce the occurrence of periodontal bone loss and gingival inflammation compared to the group of animals with experimental periodontitis and without HE treatment (*p* < 0.05). The percentage of inflammatory cells was not different comparing ligature and no-ligature groups treated with HE 125 mg/kg (*p* > 0.05) but was different in HE 250 and 500 mg/kg (Figure 4B). Interestingly, without ligature, the two highest doses of HE induced significantly more inflammatory infiltrate compared to the lowest dose of HE. Adding ligature, those same higher doses were able to significantly increase neutrophil numbers compared to those with no ligature (Figure 4D).

### 2.7. Osteoclastogenesis Was Significantly Stimulated in Bone Tissue with Ligature Independent of RANKL and Higher Concentration of HE Further Increased Osteoclast Numbers

**Figure 5:**
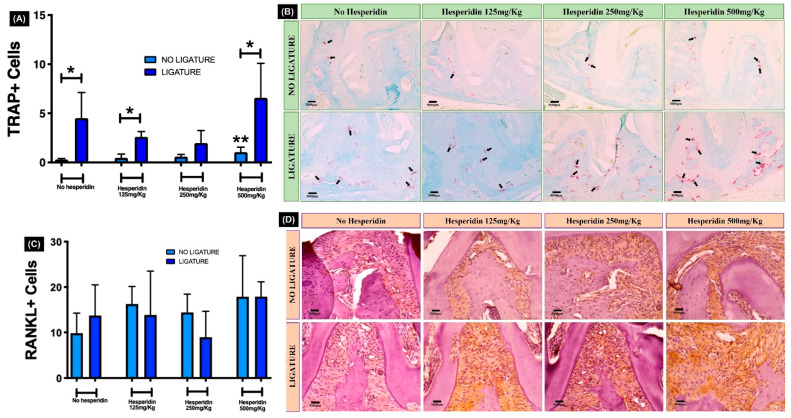
Quantification of osteoclasts in alveolar bone tissue. (**A**) Graph shows a significant increased number of TRAP+ cells in the bone tissue, generated by ligature placement. In (**B**), the black arrows indicate the multinucleated osteoclasts stained in pink (TRAP+ cells) on bone surface. * *p* < 0.05 comparing ligature and no-ligature groups; ** *p* < 0.05 compared to no-ligature and no-hesperidin group. The number of RANKL+ cells stained in brown (**D**) was not affected by ligature and/or hesperidin treatment (**C**). Photomicrographs—20× magnification.

Ligature generated a significant increased number of TRAP+ cells in the bone tissue (Figure 5A,B) compared to the no ligature. The TRAP+ multinucleated cell numbers in the ligature and no-ligature groups under HE treatment were statistically higher (* *p* < 0.05), except at 250 mg/kg. The osteoclast numbers in the ligature group were not different from no-ligature-treated mice at this concentration (corroborating the inflammatory infiltrate data). Interestingly, the highest dose of HE (500 mg/kg) resulted in a significant increase in TRAP+ cells in comparison with control, even in the absence of ligature (** *p* < 0.05). Conversely, the tissue immunoexpression of RANKL was not different between ligature and no ligature, independent of HE treatment (Figure 5C,D).

### 2.8. Systemic Dietary HE Affected Long-Term Cortical Porosity and Prevented Trabecular Wasting in Femoral Bone

**Figure 6:**
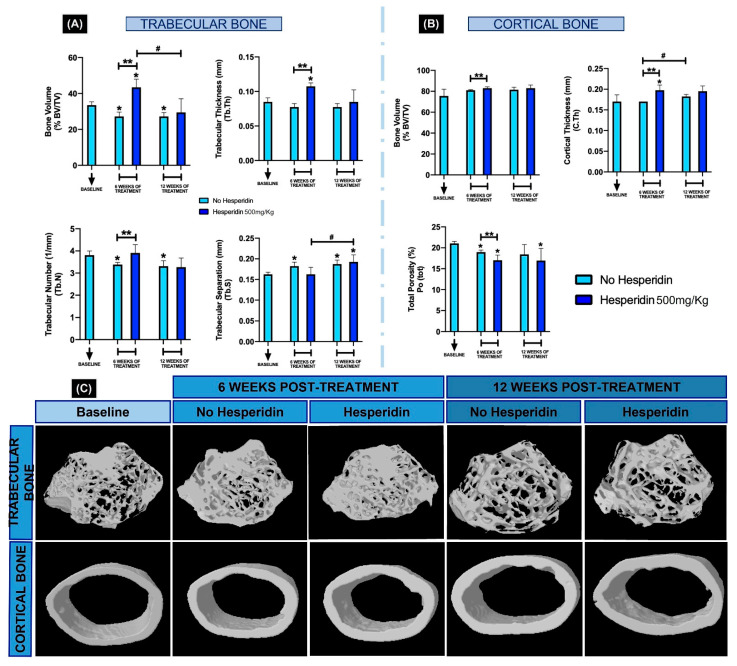
Microcomputed tomographic evaluation of femoral bone at baseline, 6 and 12 weeks post-hesperidin treatment. The microarchitecture parameters of trabecular (**A**) and cortical bone (**B**) evaluated were bone volume fraction (BV/TV, %), trabecular thickness (Tb.Th), trabecular number (Tb.N), trabecular separation (Tb.S), cortical thickness (C.Th) and cortical total porosity (Po.tot). Systemic hesperidin treatment increased BV/TV in both trabecular and cortical bone at 6 weeks, but the difference to no hesperidin was not maintained at 12 weeks. In (**C**), 3D reconstructed models of femurs show trabecular and cortical representative images per group. * *p* < 0.05 compared to the baseline group. ** *p* < 0.05 comparison between hesperidin and no hesperidin at the 6-week time point. # *p* < 0.05 comparing between 6 and 12 weeks.

Throughout the time of HE supplementation at 500 mg/kg, trabecular bone volume (BV), trabecular thickness (Tb.Th.) and trabecular number (Tb.N.) increased 6 weeks-post HE treatment (** *p* < 0.05) but returned to levels similar to baseline and no HE treatment at 12 weeks (Figure 6A,C). Overall, µCT parameters in HE-treated animals showed similar levels to no HE treatment after 12 weeks (Figure 6A,C). The volume (BV) and thickness (C.Th.) of cortical bone increased at 6 weeks of HE supplementation with reduced porosity (Po.tot.) compared to no supplementation (Figure 6B,C) (** *p* < 0.05). After 12 weeks of HE supplementation, as with the trabecular bone, the cortical parameters were not statistically significant, although the trend for HE to positively influence C.Th. remained (Figure 6B,C). In the no-HE groups at 6 weeks and 12 weeks, mice aged and showed statistically significant reduction in BV/TV, Tb.N. and increase in trabecular separation (Tb.S.) compared to baseline (Figure 6A, * *p* < 0.05).

## 3. Discussion

Phytochemicals, particularly flavonoids, are gaining increased interest for their therapeutic potential for bone. Studies reporting the effects of flavonoids in cell culture and animal models have strongly supported a role for flavonoids in bone formation and resorption in vitro and in vivo [19,22,25,27]. Additionally, the intake of flavonoids has been shown to improve bone health not only due to an influence in osteogenesis and osteoclastogenesis, but also due to their antioxidant and anti-inflammatory properties [26,27]. Therefore, due to our interest in exploring HE as a potential periodontal and bone maintenance adjuvant, our research proposed to evaluate whether HE could modulate cellular and molecular mechanisms of osteoclastogenesis in vitro and in vivo, influence inflammatory milieu and bone loss in periodontal disease and, lastly, affect long bone phenotype post-development. Three HE concentrations were proposed to evaluate the role of the dose-dependency variable in vitro and in the periodontal model, once there are no studies correlating dosage of HE with periodontal disease outcomes.

Large multinucleated bone cells (osteoclasts) differentiated from the monocyte/macrophage lineage (RAW 264.7) had significantly reduced numbers with exposure to higher dose of HE and showed a tendency for dose dependency. Corroborating this finding, Tan et al. (2017) [28] used the same in vitro protocol and evaluated the effects of neohesperidin, a hydrogenated flavonoid derivative [29], on osteoclast differentiation. The authors found that the number of differentiated cells was reduced proportionally to the increase in the compound concentration and observed impairment in cell morphology. Hesperitin, a derivate of hesperidin, was evaluated during the process of in vitro osteoclastogenesis and the compound was capable of suppressing RANKL-induced osteoclast differentiation in a dose-dependent manner in a model of LPS-induced bone loss [30]. Conversely, Yamaguchi et al. (2007) [31] evaluated the effects of different flavonoids on osteoclastogenesis in vitro and found that HE was not able to inhibit and/or reduce osteoclasts differentiation. However, it is necessary to emphasize that the authors in the latter utilized primary bone marrow cells and performed an in vitro assay for only 3 days. To assess whether the HE inhibitory effect on osteoclast differentiation was due to apoptosis, a TUNEL assay was performed, and the results showed that the number of apoptotic cells was not significantly affected by the different concentrations of HE in the first days of differentiation. The rate of apoptosis when normalized to number of cells was not different with HE 500 µM as analyzed by TUNEL. However, WB results indicated that the Caspase 3 marker showed apoptosis started to increase at day 5.

The activation of transcription factors such as c-Fos, NF-κB, and NFATc1 is required for osteoclast differentiation [32]. NFATc1 is a master transcription factor that regulates the terminal differentiation of osteoclasts, whereas c-Fos is required for osteoclast differentiation as well as bone formation [3]. The essential role of these two factors in bone homeostasis has been well documented in a series of gene disruption studies [33,34,35,36]. Protein expression of c-Fos and NFATc1 was evaluated throughout the process of cell differentiation in our study via WB analysis. We observed c-Fos protein expression in all groups during the first days of cellular stimulation with RANKL, and its suppression in the final periods of the osteoclastogenesis process. A previous report [32] describes that c-Fos is a transcription factor induced at an early stage during osteoclast differentiation, corroborating our findings. However, protein expression of c-Fos was inhibited by administration of HE at 500 µM throughout the osteoclastogenesis process, suggesting that HE at higher concentrations compromises in vitro differentiation of osteoclasts partly by inhibiting c-Fos activation. Transcription factor NFATc1 activation was also observed concomitantly with c-Fos expression on days 2 and 3 and sustained through day 5 except for HE at 500 µM which showed lower levels of NFATc1 during most of the differentiation process.. Kim and Kim (2014) [32] described that although it is unclear whether the classical or alternative NF-κB pathway is dominant in osteoclast differentiation, it is certain that the NF-κB non-canonical pathway components p50 and p65 are important for the initial induction of NFATc1 during RANKL-induced osteoclastogenesis. Further evaluation of non-canonical osteoclast activation and HE is warranted; nonetheless, our result suggests that between c-Fos and NF-κB activation changes via HE exposure, the result is a significant inhibition of osteoclast development. Our findings are comparable to the results of Liu et al. (2019) [30], who evaluated different concentrations of hesperetin and found a high dose to compromise NFATc1. Importantly, we emphasize that these results were not caused by HE cytotoxicity on cells, since protein expression of Caspase 3 was not affected.

Osteoclastogenesis involves rearrangement of the cytoskeleton structure, changes in the organelles, and the degradation and renewal of intracellular proteins [37], with autophagy implicated as a pivotal mechanism in these changes [37,38]. Autophagy has been shown to be involved in osteoclast differentiation [39,40]. Further, based on evidence that autophagy could facilitate osteoclastogenesis and osteoclastic bone resorption in RAW 264.7 cells [38], we investigated the effect of HE on autophagy in vitro. All groups, at every timepoint, except in the HE 500 µM group, showed low levels of p62 while LC3 remained evident. The higher dose of HE (500 µM) seemed to partially impair LC3-IIB conversion since there was higher expression of LC3 but no clear increase in LC3-IIB, which is important for intracellular lysosome formation and resuming autophagy. Importantly, in a normal autophagic process, the increased expression of LC3-IIB is accompanied by p62 reduction and ERK ½ phosphorylation. For the higher dose of HE, the clear overexpression of p62 throughout culture, together with no clear increase in LC3-IIB conversion despite increase in LC3 towards end of differentiation, ERK ½ phosphorylation reduction (at day 5) and a decrease in number of apoptotic cells suggest that HE may present the ability to prolong the survival of those cells; however, they are not able to sustain autophagy which can impair full osteoclast differentiation and function [40]. Li et al. (2021) [41], evaluating the potential in vitro protective effect of HE (50 µg/mL) on hypoxia/reoxygenation (H/R)-induced hepatocyte injury, found that the protein levels of autophagy markers LC3-ⅡB and Beclin-1 increased, while p62 levels decreased during H/R under exposure to HE. The authors suggested that HE induces autophagy to protect the hepatocyte against H/R injury. In our study, we found no evidence that HE improves autophagy in osteoclasts in vitro. The hepatocyte study shows some key differences, such as presence of hepatocyte injury (inflammation) and dose. It is plausible that the effect on autophagy may be dependent on cell type, environmental conditions (i.e., inflammation) and dose.

In order to evaluate the effect of HE on osteoclasts and bone homeostasis in vivo, two studies were conducted: a model of inflammatory bone loss in the alveolar housing and a skeletal bone phenotype characterization after a long (determined as enough time to affect bone connective tissue and cell function—6 weeks and 12 weeks) dietary supplementation with HE. HE has been shown to affect bone mass in rodents primarily due to inhibition of osteoclastogenesis, although there are reports that it may increase the number of osteoblasts and increase osteoblast function at low doses as well [19,22,23,25,30]. The mouse model of periodontal inflammatory bone resorption used in our study was chosen based on an acute-to-chronic inflammatory response to interdentally placed ligature [42,43] without the introduction of virulent factors such as pathogenic periodontal bacteria. This model establishes bone loss within 8 days and allows for evaluation of any potential therapeutic molecule that may act on inflammation (in the presence of commensal bacteria, not pathogenic) and bone resorption [42]. Previous studies [44,45], evaluated different experimental periodontitis models in mice and demonstrated that ligature was the most representative model for periodontal disease observed in humans. Higher TRAP+ cells on alveolar bone surface showed that osteoclast differentiation was, as expected, significantly stimulated in the ligature groups. Although it was not prevented by HE, the higher concentration (500 mg/kg) increased TRAP+ osteoclasts. Moreover, a significant increase in TRAP+ cells was observed even in the no-ligature group + HE 500 mg/kg in comparison with no ligature and no HE administration. This finding of increased osteoclastogenesis with high dose of HE alone (no ligature), combined with the finding that HE at 250 mg/kg promoted a similar inflammatory response in non-ligated mice compared with ligated mice, supports the idea that the HE effects on cells may be dependent on cell type, the HE dose and the extracellular environment. MPO levels were primarily correlated with the presence of ligature.

Correlated with osteoclast differentiation and inflammatory levels, 2D and 3D analyses of maxillae by µCT showed that the highest HE concentration (500 mg/kg) promoted a significant alveolar bone volume decrease in comparison with lower HE concentrations (125 and 250 mg/kg) when comparing all ligature groups. The bone loss in HE 500 mg/kg group was not different in animals ligated or not on HE supplementation. This finding points to a significant detrimental effect of high dose of HE on the periodontium. We highlight that 2D and 3D volumetric evaluations were consistent in detecting these results, but the linear measurement (CEJ-ABC) did not find differences in bone loss among HE treatment (4D). A previous study reporting the development of a reliable µCT methodology for quantifying tooth-supporting alveolar bone following experimental periodontitis in rats demonstrated the reliability and reproducibility of 3D µCT measurements of alveolar bone, suggesting that 3D measurements may provide better alveolar bone analysis than conventional [46].

The unaffected RANKL expression by immunohistochemistry, despite changes in osteoclast numbers with different HE doses, supports the idea that there is an alternative mechanism by which osteoclast numbers are affected by HE. Since we observed that autophagic activity is to some extent impaired by HE in osteoclasts in vitro, it is reasonable to assume that this could happen in vivo. However, impairment of autophagy has been shown to pose a negative effect on intra-oral bacterial clearance in vivo [47]. Since we observed an increased number of osteoclasts and alveolar bone resorption with a high dose of HE in vivo (even independent of ligature presence), there are a few possible scenarios that may explain this finding: (1) HE may have stimulated the observed high level of bone resorption in vivo as a result of the effect of a pro-oxidant activity creating a toxic environment in an exacerbated inflammatory milieu (as reported for other flavonoids given in high doses) [48]; (2) HE’s influence on impairment of autophagy can lead to bacterial persistence on site, especially at higher doses of HE [47]. In long bones not subjected to the effect of bacteria and with a very different redox state (no injury or ligature present) around the cell environment, the response of bone to a high dose of dietary HE could differ. Indeed, other studies have shown that systemically administered HE leads to an increase in bone mass in rats, which is the opposite to that which was observed in our maxillae study [19,22].

A study that investigated the effects of HE on experimental periodontal disease in rats demonstrated that the compound prevented the progression of ligature-induced bone loss and effectively inhibited the expression of gingival proinflammatory cytokines [26]. Supporting our thoughts on the differences between flavonoid intake regimen in tissue response, the HE treatment was given just concomitantly to ligature placement for 7 days (not 4 weeks before). Further, the treatment with the compound was performed via oral intake on methylcellulose vehicle, possibly promoting retention in the oral cavity and a local action of HE on the periodontal tissues and bacteria resulting in limited oral systemic intake. Lastly, the highest concentration of HE used was 150 mg/kg, basically equivalent to our lowest concentration in the study but used for only 1/6 of the time. In another study, Liu et al. (2019) [30] evaluated the effect of the derivative hesperitin in a mouse model of LPS-induced femur bone resorption. It was shown that in the animals treated with hesperitin the percentage of bone volume of femurs was significantly higher than in those animals that received only LPS injection and the number of osteoclasts was fewer when HE was administered with LPS. LPS and HE were given intraperitoneally at a 5 mg/kg and 100 mg/kg doses, respectively. As highlighted above, it is important to discuss the regimen in the context of different cells and tissue conditions to appropriately interpret these studies.

Greabu et al. (2020) [49] described the molecular path towards periodontitis initiation and progression presents four key steps: bacterial infection, inflammation, oxidative stress, and autophagy. The same study concluded that in the context of periodontitis, excessive reactive oxygen species (ROS) generation triggers intensive inflammatory reactions, apoptosis, and disturbs autophagy activity, inducing periodontal tissue alterations [49]. Indeed, previous studies showed that in periodontal ligament tissues, in patients with periodontal disease, the expression of LC3 is significantly upregulated compared to healthy patients [50], and the areas of resorbed alveolar bone also showed higher levels of autophagy in a mouse model of periodontitis [51,52]. Autophagy is a necessary mechanism of bacterial clearance and, if disturbed, may be a risk indicator for periodontitis [47]. Therefore, based on our in vitro osteoclast assay and the in vivo results from alveolar bone resorption along with the high inflammatory infiltrate with highest doses of HE, we propose that decreased autophagy in the presence of HE (and, potentially, defects in bacterial clearance and an associated increase in redox status), can lead to the exacerbated bone loss observed in the presence of high doses of the flavonoid HE. Our future work will include validation of this hypothesis on bacteria and ROS levels.

In alignment with this line of thought, the response of long bones to a high dose of HE in the absence of injury or external inflammatory triggers was positive, presenting as an increase in bone mass parameters. The encouraging effect of lower doses of HE systemically delivered in long bones is supported by evidence demonstrating that a diet containing HE 0.5% prevents androgen-deficiency-induced bone loss in male mice [53], inhibits bone loss in ovariectomized mice [23] and neohesperidin ameliorates steroid-induced osteonecrosis of the femoral head mice model [54], and suppresses osteoclast differentiation, bone resorption and ovariectomized-induced osteoporosis in mice [28]. Our results evaluating the effects of high HE concentration (500 mg/kg) on femur phenotype showed that systemic HE intake reduced the cortical bone porosity and prevented a trabecular bone volume decrease, which supports the idea that in our periodontal model, the complexity of the disease environment and the local inflammation were crucial factors to modify the cell responses to HE. However, the positive 6-week effect of HE intake on the trabecular and cortical bone parameters were not sustained after 12 weeks. There may be metabolic changes taking place during long-term treatment with bone sparing molecules (i.e., such as in the case of parathyroid hormone therapy) to overcome bone homeostasis effects induced by HE [55]. Thus, further studies on administration protocols such as intermittent vs. continuous, different doses and delivery systems are warranted to best utilize HE as a long bone anabolic molecule. The femur model was limited to assessment of the tomographic parameters; thus, it is not possible to conclude if the beneficial effects of HE at 6 weeks of intake were due to tissue osteoclastogenesis suppression either associated or not with a stimulatory effect on osteoblasts and bone formation and any ROS effect. Therefore, mechanistic investigations are further required. In addition, consideration of age, bone maturity and sex need to be evaluated within HE supplementation context. From this perspective, a previous study investigated the bone-sparing effect of HE in young and adult ovariectomized female rats and observed a clear protective effect of HE on bone loss and strength in ovariectomized rats, but the older rats were more sensitive to the ovariectomy-induced bone loss than the younger rats and the main effect of HE was a decrease in bone resorption [56]. Moreover, on one hand, HE stimulated bone mineral density in young intact rats and on the other hand, femoral strength in older intact rats, emphasizing the HE effects’ dependence on bone maturity [56].

Taken together our results showed a modulatory effect of HE on osteoclastogenesis, inflammation, periodontal disease severity and skeletal bone homeostasis in mice. Our study challenges previous work evaluating HE as an adjuvant for promoting periodontal health, as our results clearly demonstrate a non-beneficial modulation of high concentrations of HE on inflamed and non-inflamed periodontal tissues. The overall idea that flavonoids are primarily beneficial for bone maintenance needs to be further clarified, including emphasizing the effect of dosage and tissue environment.

In conclusion, HE impaired in vitro osteoclastogenesis in RAW 264.7 cells and seems to involve at least both c-Fos and NFATc1 downstream of the NF-κB pathway as well as a negative modulatory response of autophagy. Oral administration of a high concentration of dietary HE, however, promoted increased osteoclast numbers and potentiated insult-induced alveolar bone loss due to an inflammatory and potentially hyper oxidative environment. On the other hand, in accordance with previous literature, HE can present a bone-sparing effect on skeletal bone homeostasis under physiological conditions. Further mechanistic investigations are currently underway for thorough characterization of HE effects in bone.

## 4. Materials and Methods

### 4.1. Cell Culture and Hesperidin Dilution

RAW 264.7 cells were purchased from American Type Culture Collection (Manassas, VA, USA) and were grown in α-minimum essential medium (DMEM, Gibco, Carlsbad, CA, USA), containing 10% fetal bovine serum (Corning, Corning, NY, USA) and supplemented with penicillin/streptomycin 1% and glutamax 1%, in a 5% CO_2_ atmosphere at 37 °C.

Concentrations of HE (ACROS Organics, Geel, Belgium) of 1 µM, 100 µM and 500 µM were used. HE stocks solutions were prepared after initial dilution in pure DMSO (99.5%). At each treatment timepoint, the final concentrations of HE were prepared from stock solutions in cell culture medium, containing fetal bovine serum 10% (Corning), penicillin/streptomycin 1% and glutamax 1%. 0.5 µL of HE stock solutions (1 M, 200 mM and 2 mM) were added into each 1 mL of cell culture medium, resulting in 0.05% final concentration of DMSO.

### 4.2. RAW 264.7 Cell Differentiation and Apoptosis In Vitro Assay

RAW 264.7 cells were plated onto 96-well plates at a density of 3.2 × 10^3^ cells/well in medium, as described above. The cells were treated with HE at 1, 100 or 500 µM in 0.05% DMSO, differentiation medium alone (no HE), or no RANKL (negative control). Cell differentiation was stimulated by 30 ng/mL RANKL (R&D Systems, Minneapolis, MN, USA) of cell culture medium. The HE treatments, as well as RANKL, were added on day 1. The cells were stimulated for 5 days, refreshing culture medium and treatments every two days. To assess the cell differentiation, at the end of the fifth day, the supernatant of each well was aspirated, and the cells were immediately washed with sterile phosphate-buffered saline (PBS) (1×) and then fixed using 10% formaldehyde. After fixation, cells were washed again with PBS and in each well Tatrate Resistant Acid Phosphatase (TRAP) solution (containing—Sodium Acetate Anhydrous, Sigma S-2889/L-(+) Tartaric Acid, Sigma T-6521/Distilled Water/Glacial Acetic Acid, Sigma 695092/Fast Red Viole LB Salt, Sigma F-3381/Naphthol AS-MX Phosphate Substrate mix, Sigma N-4875) (Sigma, St.Louis, MO, USA) was added for 10 min at 37 °C, then the wells were washed with distilled water. Subsequently, the cells on the surface of the wells were photographed and quantified using an EVOS microscope (EVOS XL Core Imaging System, Life Technologies Corporation, Bothell, WA, USA). Osteoclasts were identified as cells stained red/pink with three or more nuclei. For apoptosis, the Terminal deoxynucleotidyl transferase dUTP (TUNEL) protocol Trevigen HT TiterTACS Kit (Trevigen Inc, Minneapolis, MN, USA) was used according to the manufacturer’s recommendations and absorbance measured on the Cytation 5 equipment (BioTek™, Winooski, VT, USA) at a wavelength of 450 nm. Each assay was repeated in triplicate.

### 4.3. Western Blotting Analysis

RAW 264.7 cells were plated onto 100 mm dishes at a density of 11 × 10^5^ cells/dish, in α-minimum essential medium (α-MEM, no phenol red, Gibco) as described above. The cells were treated with HE at 1, 100 or 500 µM in 0.05% DMSO and combined with 30 ng/mL RANKL (R&D Systems) to stimulate differentiation. The treatments, as well as RANKL, were added on day 1. The in vitro osteoclastogenesis was stimulated for 5 days, refreshing culture medium and treatments every two days. On days 2, 3, 4 and 5 of the assay, the supernatant of each dish was aspirated and cells harvested. Briefly, RIPA Buffer solution (ThermoFisher Scientific, Waltham, MA, USA) containing protease inhibitors was added into the plates and using a scraper, the cells were detached and transferred to Eppendorf tubes and kept at −80 °C. Protein quantification was performed using the BCA Protein Kit (Pierce BCA Protein Kit, Thermo Fisher Scientific, Waltham, MA, USA) according to the manufacturer’s guidelines and samples were prepared for Western Blotting (WB) to evaluate the protein expression of c-Fos (Cat. #4384s, Cell Signaling, Danvers, MA, USA), NFATc1 (Cat. #sc-7294, Santa Cruz Biotechnology, Dallas, TX, USA), Caspase 3 (Cat. #9662s, Cell Signaling), p62 (Cat. #5114s, Cell Signaling), LC3 (Cat. #2775S, Cell Signaling) and as a control, β-actin 1 (Cat. #sc-1616, Santa Cruz Biotechnology). In total, 5–10 µg of the total protein lysate was resolved using Criterion TGX precast gel (Biorad, Hercules, CA, USA) and transferred to a nitrocellulose membrane using the Trans-Blot Turbo Transfer System (Biorad) and immunodetected using appropriate primary and peroxidase-coupled secondary antibodies (Goat-Anti-Rabbit IgG, ThermoFisher #31460). Proteins were visualized using enhanced chemiluminescence (ECL, Amersham Bioscience, Chicago, IL, USA). The protein levels were evaluated relative to β-actin via use of Image J (NIH, Bethesda, MD, USA) [57,58].

### 4.4. In Vivo Periodontitis Model and Long Bone Phenotyping

#### 4.4.1. Animals

The animal experiment protocol was approved by the Institutional Animal Care and Use Committee (IACUC) at the University of North Carolina at Chapel Hill (IACUC ID: 18–115, 23 May 2018). The procedures were also conducted in compliance with the Brazilian National Council for the Control of Animal Experimentation, with ethical standards that fully comply with Animal Research: Reporting of In Vivo Experiments (ARRIVE) guidelines.

Sixty male C57BL/6 mice at 6 weeks of age (20–27 g) were kept in an environment with controlled temperature, humidity, and light cycles, and fed with water and feed ad libitum. Forty mice were subjected to HE oral gavage starting at 7 weeks of age and ligature-induced periodontitis starting at week 11. Twenty mice started dietary HE supplementation via oral gavage at 7 weeks of age throughout 19 weeks of age for the evaluation of long bone phenotype. Sex as a biological variable was not investigated in the study to avoid the influence of the variable estrous in periodontitis and bone phenotype (Supplementary Figure S1).

#### 4.4.2. Periodontal Disease Model

Forty mice were randomly distributed in the following groups: (1) control group (no HE), (2) HE 125, (3), HE 250 and (4) HE 500 mg/kg (*n* = 10/group). All four groups were challenged with the periodontal ligature model after 4 weeks of HE gavage and continued dietary HE supplementation through the ligature challenge. HE (97% purity, ACROS Organics) concentrations, at 125, 250 and 500 mg/kg of mice weight, were diluted in 0.9% sodium chloride and administered once daily by oral gavage until euthanasia [Supplementary Figure S1A]. The HE effects associated with an experimental periodontal disease model were firstly published in 2019 [26], and the authors reported an ameliorative and dose-dependent effect of HE (at 75 and 150 mg/kg concentration) on ligation-induced periodontitis in rats. Based on this study, we aimed to investigate higher HE concentrations on ligature-induced periodontitis to understand how low, intermediate and high HE concentrations compare in their bone-response effect. Toxicity with higher doses was not a concern, as supported by a previous study [59] evaluating the therapeutic benefits of HE at 125, 250 and 500 mg/kg using a psoriasis-like mouse model. Animal weight gain and distress were monitored throughout the study to ensure animal welfare.

Briefly, for the ligature placement, the animals were anesthetized using isoflurane under sterile conditions. The animal was secured in a proper mouse dental bed to facilitate isofluorane delivery and opening of the mouth using rubber bands. A sterile silk suture (Roboz Surgical Instrument, SUT-15-1, Gaithersburg, MD, USA) was placed between the 1st and 2nd right maxillary molars [42]. The ligature was kept in place for 10 days and was checked every day for possible displacement. The left hemimaxilla was set as negative control (no ligature placement). The mice were euthanized by cervical dislocation at day 10, preceded by CO_2_ inhalation as approved by IACUC, and the maxillae were harvested and fixed in 10% buffered formalin for 48 h. The samples were analyzed using microcomputed tomography (µCT) using micro-focus X-ray CT system scanner (Skyscan 1275, Billerica MA, USA), followed by demineralization with 0.5 M EDTA at pH 7.4 for 6 weeks, paraffin embedding and histopathological processing.

#### 4.4.3. Femoral Phenotyping Study

Twenty mice (*n* = 4/group) were distributed into a Baseline group (7 weeks of age) (1), a group treated (2) or not (3) treated with dietary HE 500 mg/kg for 6 weeks (13-week-old mice), and a group treated (4) or not (5) treated with HE 500 mg/kg for 12 weeks (19-week-old mice). HE at 500 mg/kg of mice weight was diluted in 0.9% sterile sodium chloride and administered daily, once a day by oral gavage, starting from Baseline until the euthanasia day at week 6 or week 12 post start of the gavage. Animals from Baseline group were euthanized on week 0 (bone phenotype at 7 weeks of age). The euthanasia was performed by cervical dislocation, preceded by CO_2_ inhalation, and the femurs were harvested. The samples were fixed in formalin 10% for 48 h and processed for µCT analysis (Supplementary Figure S1B).

#### 4.4.4. Microcomputed Tomography Bone Analysis

The hemimaxillae were scanned using a microcomputed tomography (µCT) system (Skyscan 1275, Bruker) at 12.5 µm, 70 kVp, 145 µA using 1 mm aluminum attenuation filter. The three-dimensional images were reconstructed using the software NRecon 1.6.9.8 (SkyScan), Data Viewer 1.5.0 (SkyScan) and CTAnalyzer (CTAn—2003-11SkyScan, 2012 Bruker MicroCT, 1.13.11.0 version) and were used for image re-orientation and analysis, respectively. For each sample, a standardized region of interest (ROI) was drawn covering alveolar bone from the distal surface of the second molar to the mesial surface of the first molar, including buccal/lingual faces. In this standardized ROI, bone volume fraction was determined using a binarization method with standardized threshold to differentiate mineralized and non-mineralized tissues. Mineralized tissue percentage corresponding to the root was subtracted from the bone volume fraction and therefore the bone volume/total volume (BV/TV) fraction of the ROI was calculated [60]. We assessed bone loss by measuring the cemento–enamel junction to alveolar bone crest (CEJ-ABC) distance on the distal, medial and mesial side of the first and second molars on each of the buccal surfaces [42].

For the femurs, the Skyscan 1275 scanner was used at 12.45 µm, 70 kVp, 142 µA using a 0.5 mm aluminum attenuation filter. The ROI included a set of specific number of slices below the growth plate after a set anatomical marker for trabecular bone and cortical bone analysis (50 and 400 slices below growth plate, respectively). The bone microarchitecture parameters evaluated were trabecular and cortical bone volume fraction (BV/TV, %), trabecular thickness (Tb.Th), trabecular number (Tb.N), trabecular separation (Tb.S), cortical thickness (C.Th) and cortical total porosity (Po.tot) as the recommended parameters reported by Bouxsein et al. (2010) [61].

#### 4.4.5. Histopathological Analysis of Periodontal Tissues

The EDTA-demineralized hemimaxillae were embedded in paraffin in a standardized spatial orientation to obtain 6 µm thick serial sections on its sagittal plane. The sections were mounted on glass slides and stained with H&E for histomorphometric evaluation and stereometry, TRAP for osteoclast numbers assessment and immunohistochemistry for RANKL for correlation to osteoclast numbers, and myeloperoxidase (MPO) for neutrophil numbers. The stained sections were photographed using an EVOS microscope (EVOS XL Core Imaging System, Life Technologies Corporation, Carlsbad, CA, USA).

Qualitative and stereometric analyses of periodontal structures were performed from H&E-stained images. For stereometry, a 32,400 µm^2^ grid with 9 × 4 µm squares was constructed and overlayed on the digital images obtained from at least 15 histological sections. The region of interest for the analysis was represented by the whole grid, which was positioned in a submarginal area of the gingival interproximal surface. Stereometric analysis using a point-counting technique for the inflammatory cells observed on each intersection point of the grid was performed by an experienced blinded observer [60].

Non-serial histological sections were stained for TRAP+ multinucleated osteoclasts. Briefly, the slides were deparaffinized and rehydrated through graded ethanol to distilled water, and then immersed in TRAP staining solution mix (at 37 °C for 30 min). The sections were rinsed in distilled water and counterstained with 0.02% fast green for 30 s. For the quantification, the TRAP+ cells stained in red, containing three or more nuclei on the bone surface, were considered osteoclasts and quantified in the ROI comprising the furcation area of the first and second molars and in the interproximal area between the two teeth.

For immunohistochemistry, to assess the immunoexpression of RANKL and MPO [62], an antigen retrieval was performed on decalcified sections using a citrate buffer. After overnight incubation with the primary antibodies (RANKL—Abcam 216484, Cambridge, UK, 1:750; MPO—Abcam 208670, 1:1000), sections were incubated with the universal biotinylated secondary antibody (Biotinylated link—K0675 Dako Kit, Agilent, Santa Clara, CA). The target protein was detected using a DAB-streptavidin system (LSAB-2, Dako Cytomation). The sections were counter-stained with hematoxylin for 45 s, and coverslips were mounted in Permount^TM^ Mounting Medium (Fisher Chemical^TM^, Fair Lawn, NJ, USA). The RANKL-positive cells stained in brown were quantified in the ROI as described above for TRAP [63].

### 4.5. Statistical Analysis

For all in vitro studies, three independent experiments were performed. For both, in vitro and in vivo studies, data were submitted to the Kolmogorov–Smirnov or Shapiro–Wilk tests to assess homogeneity and data distribution. Analysis of variance (ANOVA) with Tukey’s post hoc test and Kruskal–Wallis with Dunn post hoc were performed to determine the differences among groups of animals. A Student’s *t* test or Mann–Whitney test was used to evaluate meaningful differences among ligated or non-ligated animals with and without HE exposure. All analyses were performed using GraphPad Prism 8 software (GraphPad Software Inc., San Diego, CA, USA). All tests were applied with a 95% confidence level (*p* < 0.05). Data were expressed as mean ± standard deviation.

## 5. Conclusions

In conclusion, HE impaired in vitro osteoclastogenesis, but on the contrary, oral administration of a high concentration of dietary HE increased osteoclast numbers and promoted inflammation-induced alveolar bone loss. However, HE at 500 mg/kg can promote a bone-sparing effect on skeletal bone under physiological conditions. This work highlights the importance of studies considering careful evaluation of the role of phytochemicals on bone health and other tissues based on dose and tissue microenvironment. Further, this study focused on one natural flavonoid only; thus, the effect of commonly used phytochemicals such as other polyphenols, carothenoids, thyosulfates and pro/pre-biotics in relationship to the variables investigated is warranted.

## Data Availability

Any raw data supporting reported results can be requested by contacting the corresponding author.

## Supplementary Materials

The following supporting information can be downloaded at: https://www.mdpi.com/article/10.3390/ijms23137100/s1.
