# Supplementary material for: Systemic Dietary Hesperidin Modulation of Osteoclastogenesis, Bone Homeostasis and Periodontal Disease in Mice
Source: Int J Mol Sci. 2022 Jun 26;23(13):7100. doi: 10.3390/ijms23137100 (PMC9266620; doi:10.3390/ijms23137100)
Supplement: Supplementary file 1 [file ijms-23-07100-s001.zip › ijms-1799347-supplementary.pdf]

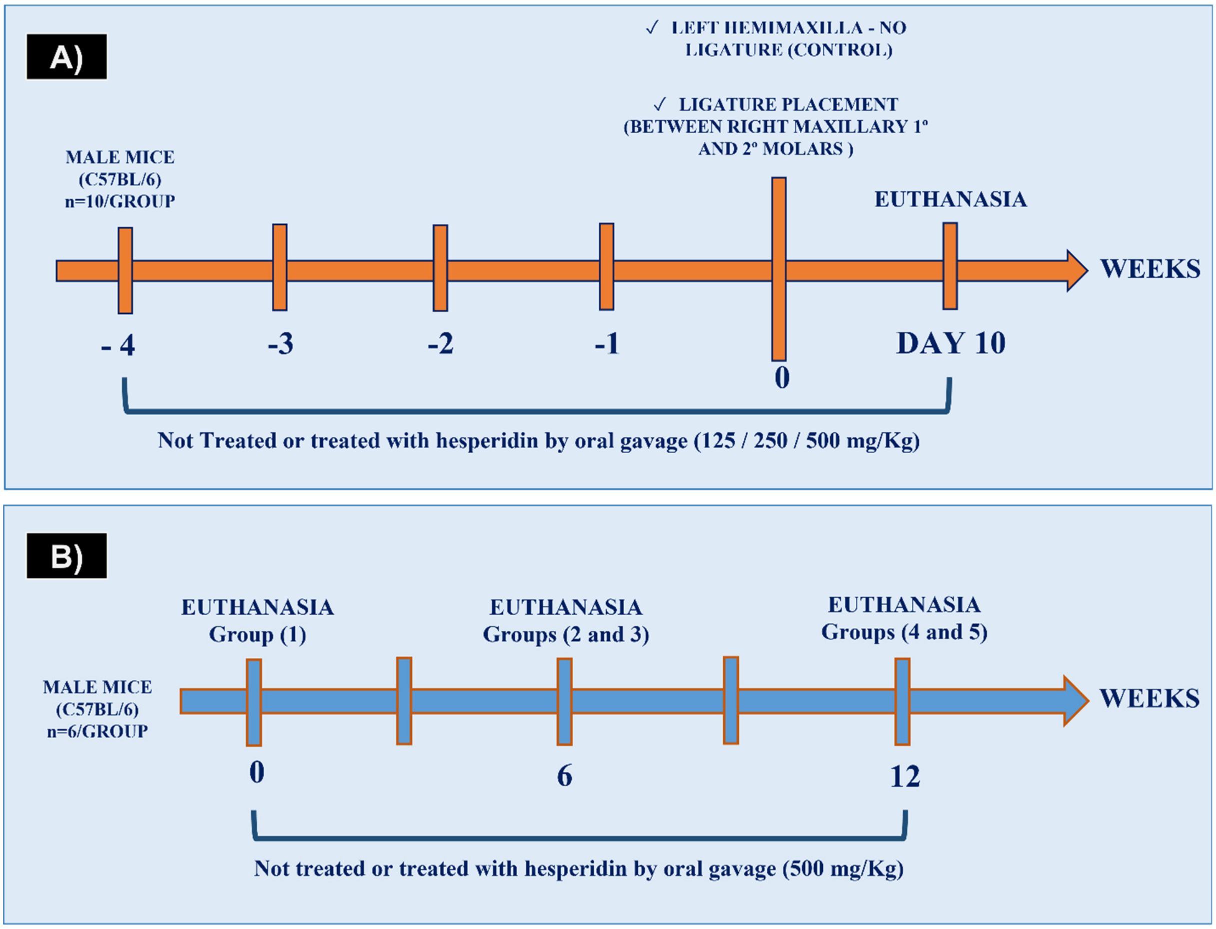

Figure S1. Ligature placement or systemic HE administration did not promote any behavioral change, physical issues (e.g., skin lesions, hair loss), detrimental weight loss or feeding impairment
